# Study on the Potential Mechanism of Fructus Tribuli in the Treatment of Hypertensive Vascular Remodeling Based on Network Pharmacology and Molecular Docking

**DOI:** 10.1155/2021/8862176

**Published:** 2021-01-08

**Authors:** Shuyue Wang, Fei Guo, Xiaochen Sun, Xiao Song, Yaohui Yuan, Chao Zhang, Guitao Lin, Huagang Sheng

**Affiliations:** ^1^School of Pharmaceutical Sciences, Shandong University of Traditional Chinese Medicine, Jinan 250355, China; ^2^The Affiliated Hospital of Shandong University of Traditional Chinese Medicine, Jinan 250011, China

## Abstract

**Background:**

Hypertensive vascular remodeling (HVR) is the pathophysiological basis of hypertension, which is also an important cause of vascular disease and target organ damage. Treatment with Fructus Tribuli (FT), a traditional Chinese medicine, has a positive effect on HVR. However, the pharmacological mechanisms of FT are still unclear. Therefore, this study aimed to reveal the potential mechanisms involved in the effects of FT on HVR based on network pharmacology and molecular docking.

**Materials and Methods:**

We selected the active compounds and targets of FT according to the Traditional Chinese Medicine Systems Pharmacology Database and Analysis Platform (TCMSP) and the Swiss Target Prediction database, and the targets of HVR were collected from the Online Mendelian Inheritance in Man (OMIM), GeneCards, and DrugBank databases. The protein-protein interaction network (PPI) was established using the STRING database. Moreover, Gene Ontology (GO) and Kyoto Encyclopedia of Genes and Genomes (KEGG) pathway analyses and network analysis were performed to further explore the potential mechanisms. Finally, molecular docking methods were used to evaluate the affinity between the active compounds and the main target.

**Results:**

Seventeen active compounds of FT  and 164 potential targets for the treatment of HVR were identified. Component-target and PPI networks were constructed, and 12 main active components and 33 main targets were identified by analyzing the topological parameters. Additionally, GO analysis indicated that the potential targets were enriched in 483 biological processes, 52 cellular components, and 110 molecular functions. KEGG analysis revealed that the potential targets were correlated with 122 pathways, such as the HIF-1 signaling pathway, ErbB signaling pathway, and VEGF signaling pathway. Finally, molecular docking showed that the 12 main active components had a good affinity for the top five main targets.

**Conclusion:**

This study demonstrated the multiple compounds, targets, and pathway characteristics of FT in the treatment of HVR. The network pharmacology method provided a novel research approach to analyze potential mechanisms.

## 1. Introduction

Hypertension has become one of the most threatening public health problems worldwide [1]. It is the leading cause of death from cardiovascular and cerebrovascular diseases. Hypertensive vascular remodeling (HVR) is an adaptive change in the arterial structure and function. It is a self-protective response to trauma and internal and external pressure changes to meet the physiological needs under different pathological conditions [2]. It mainly includes thickening of the vessel wall, a change in the wall-to-cavity ratio, and looseness of the small artery structures, resulting in abnormal vascular function. Basic and clinical studies on hypertension showed that vascular remodeling was accompanied by hypertension, was the pathophysiological basis [3], and formed a vicious circular causal mechanism with hypertension, which was also an important cause of vascular disease and target organ damage [4]. Reducing or reversing vascular remodeling has important theoretical significance and potential clinical application value for the early prevention and treatment of hypertension and its cardiac, brain, and renal complications [5, 6]. Therefore, it is particularly important and urgent to search for targets that affect the vascular structure and research and discover therapeutic drugs that are safe and effective in controlling HVR.

Traditional Chinese medicine (TCM) has high activity and low toxicity in preventing and treating HVR, playing an irreplaceable role. Fructus Tribuli (FT), which is the dried fruit of *Tribulus terrestris* L. [7], is renowned in TCM as Ji-Li and has been used for thousands of years in Asian countries. Several previous investigations focused on its pharmacological activities demonstrated that FT improved sexual function [8], could be used to prevent and treat cardiovascular diseases [9], had neuroprotective [10] and memory improvement activity [11], and provided antidiabetic [12], antidepression [13], anti-inflammatory [14], and antioxidant effects [15].

In recent years, the efficacy of FT in HVR has attracted more and more attention. Zhang et al. found that the furostanol saponins from FT could inhibit the expression of myocardial transforming growth factor and connective tissue growth factor in ventricular muscle, reduce systolic blood pressure, and inhibit myocardial fibrosis in spontaneously hypertensive rats (SHRs). Thus, vascular remodeling induced by hypertension was significantly ameliorated [16]. Guo et al. showed that FT could reduce blood pressure and inhibit aortic vascular remodeling in elderly SHRs. The primary mechanisms involved are thought to be reduced collagen content in the thoracic aorta and regulation of the NF-*κ*B signaling pathway [17]. Jiang et al. found that FT demonstrated antihypertensive and endothelial protective effects by regulating Erk2, FAK, and NF-*κ*B p65 [18]. In addition, several clinical studies demonstrated that the Chinese patent medicine Xin-Nao-Shu-Tong, with extracts of FT as the active compounds, had a reliable antihypertensive effect [19]. However, its mechanism has not been fully elucidated. Thus, further comprehensive and systematic investigations are necessary.

TCM has the characteristics of integrity and diversity and can play a comprehensive role in multilevel, multilink, multitarget, and bidirectional regulation. Network pharmacology is a new field that integrates systems biology, omics, and computational biology to reveal the mechanism of drug action from an overall perspective and possesses integrity, synergistic effects, and dynamic characteristics [20]. This research thinking is consistent with the characteristics of the simultaneous effects of the multiple components of TCM [21]. Therefore, this study aimed to reveal the possible molecular mechanisms involved in the effects of FT on HVR based on a network pharmacology approach.

## 2. Materials and Methods

### 2.1. Collection of Active Compounds of FT

The chemical information about FT was obtained from the Traditional Chinese Medicine Systems Pharmacology Database and Analysis Platform (TCMSP, https://tcmspw.com/tcmsp.php), which is considered a special platform for Chinese herbal medicine based on systems pharmacology that provides the relationships between drugs, targets, and diseases, as well as comprehensive data on absorption, distribution, metabolism, and excretion (ADME) properties for each compound [22]. To screen for active compounds, oral bioavailability (OB) ≥ 30% and drug-likeness (DL) ≥ 0.18 included in the ADME system were selected as the screening criteria by referring to the most common criteria in the TCMSP database [23, 24]. In addition, by consulting the literature, we added several active compounds, which were removed by ADME screening or not recruited by the TCMSP database but have some potential value in the treatment of HVR. Then, the primary molecular formula of all active compounds was double-checked in the PubChem database (https://pubchem.ncbi.nlm.nih.gov/).

### 2.2. Screening of Targets Related to Active Compounds

The TCMSP and Swiss Target Prediction (http://www.swisstargetprediction.ch/) database were applied to identify the potential targets related to the active compounds of FT. Swiss Target Prediction is a web server for potential drug target prediction. Then, each of the predicted targets was inputted into the UniProt database (https://www.uniprot.org/) with “Homo sapiens” selected as the organism. After deleting repeated and nonhuman targets, we obtained the targets for each active compound.

### 2.3. Screening of Targets for HVR

Targets associated with HVR were retrieved from the Online Mendelian Inheritance in Man (OMIM, http://www.omim.org/) database, the GeneCards database (https://www.genecards.org/), and the DrugBank database (https://www.drugbank.ca/). OMIM is a comprehensive research resource of human genes and genetic diseases [25]. GeneCards is an integrative database, which provides information on all annotated and predicted human genes [26]. The DrugBank is a powerful bioinformatics database containing information on drugs, diseases, and their mechanisms of action and targets [27]. All of these databases are comprehensive, freely available online tools and can provide a relatively comprehensive overview of research results.

### 2.4. Construction of the Target Protein-Protein Interaction Network

To identify the potential targets of FT in the treatment of HVR, we intersected the active compound targets and disease targets. The common targets were considered to be potential targets. The obtained common targets were imported into STRING (https://string-db.org, Version 11. 0) to perform protein-protein interaction (PPI) analysis. STRING is a database that predicts direct and indirect interaction and builds networks between proteins and proteins [28]. The data were saved in a tab-separated value (TSV) format and imported into Cytoscape 3.6.1 software where the PPI was drawn and analyzed [29]. We calculated the topological parameters of the network using the plug-in of “Network Analyzer.” We then used degree, betweenness, and closeness as the three main parameters for critical target screening [30]. The Molecular Complex Detection (MCODE) plug-in was used to screen the densely connected regions and for cluster analysis of the PPI network [31]. We selected significant cluster modules from the constructed PPI network using MCODE. The degree of protein association in the module was scored using the following criteria: degree cutoff = 2, node score cutoff = 0.2, K-score = 2, and max depth = 100.

### 2.5. Gene Ontology and Pathway Analysis

All potential targets were utilized for Gene Ontology (GO) enrichment analysis and Kyoto Encyclopedia of Genes and Genomes (KEGG) pathway enrichment analysis using the DAVID database (https://david.ncifcrf.gov/, Version 6.8). DAVID can provide systematic, comprehensive biological functional annotations for large-scale gene or protein lists, including biological processes (BP), cellular components (CC) molecular functions (MF), and pathways. In the GO and KEGG analyses, the lower the *P* value, the stronger the correlation between the pathway and target; the higher the count, the more targets in the pathway. A threshold *P* value of <0.05 was considered statistically significant.

### 2.6. Construction of the Compound-Target-Pathway Network

To further explore the pharmacological mechanisms of FT  in the signaling pathway for treating HVR, the predicted target genes of each compound, the disease-related genes, and the KEGG pathway involved were all imported into Cytoscape software to establish a combinatorial network. In the network, the nodes represent compounds, targets, and pathways, while the edges represent the interaction between the nodes.

### 2.7. Molecular Docking

The molecular structures of the major active compounds in the Mol2 file format obtained from the TCMSP in the simulation description format (SDF) downloaded from the PubChem database or in the canonical simplified molecular-input line-entry specifications (SMILES) were imported into the Discovery Studio Client software for modification. The RCSB PDB database (http://www.rcsb.org/) was searched to download the major target protein structure and import it into Discovery Studio Client for preprocessing such as deleting the water molecules and preparing the protein. The original ligand position was used as the active center to expand a certain range, and the amino acid residues in this range were defined as the active site. The default settings were used for the other settings. The receptor-ligand interaction module was used for molecular docking. The software finds the optimal binding conformation by locating the active sites of small molecule compounds and proteins and calculates and scores the affinity between the receptors and the ligands. LibDock scores between the compound and protein higher than the docking score between the ligand and the protein indicate that the component has a good affinity for the target. Finally, PyMOL software was used to visualize the docking results of the major active compound with their corresponding higher-scoring protein.

## 3. Results

### 3.1. Screening of Active Compounds and Related Targets

Fifty-one compounds were screened by searching the TCMSP. Twelve compounds were collected using the screening conditions of OB ≥ 30% and DL ≥ 0.18. Furthermore, previous studies reported that steroidal saponins are one of the main compounds of FT, which can be used to treat cardiovascular diseases [32, 33], so we added several steroidal saponins that were deleted because of the screening conditions or not recruited by the TCMSP. Finally, 17 active FT compounds were selected, which are shown in Table 1. The targets related to active compounds were predicted by the TCMSP and Swiss Target Prediction database. After combining the retrieval results and removing the duplicated targets, a total of 164 potential targets were obtained.

### 3.2. Screening of Targets of FT in the Treatment of HVR

**Figure 1:**
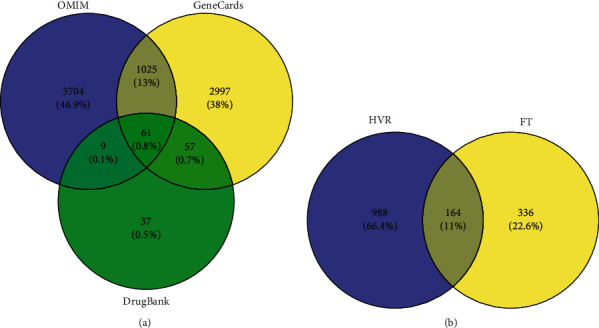
Venn diagram of related targets of FT in treating HVR. (a) The purple circle represents the targets of HVR from OMIM. The yellow circle represents the targets of HVR from GeneCards. The green circle represents the targets of HVR from DrugBank. (b) The purple circle represents the targets of HVR. The yellow circle represents the targets of the active compounds from FT.

In this study, we used three internationally recognized databases of disease genes. To improve the accuracy of the targets, the three database results were intersected using the Venny platform [34]. A total of 1152 targets were retrieved, as shown in Figure 1(a). By intersecting the target of HVR with that of FT, a total of 164 overlapping target genes were identified, which we considered potential targets of FT for treating HVR, as shown in Figure 1(b).

### 3.3. Construction of the Compound-Target Interaction Network

**Figure 2:**
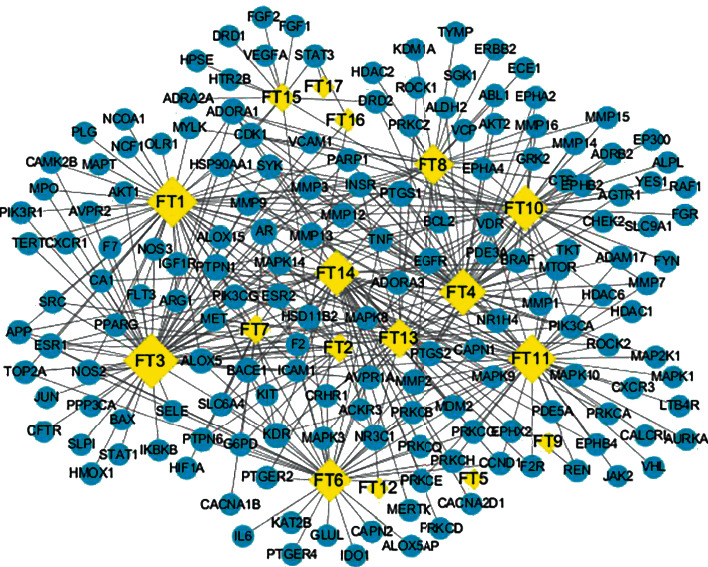
Compound-target interaction network. The yellow nodes represent the compounds, the blue nodes represent the targets, and the edges represent the interaction between them.

The interactions of the abovementioned 17 active compounds and 164 potential targets were defined as data and type files in Excel file format, and the relationship was visualized by Cytoscape software. Then, we constructed the compound-target interaction network, as shown in Figure 2. The diamonds represent the active compounds, and the circles represent their targets. A total of 181 nodes and 425 edges were obtained. The network topological parameters were analyzed using the Network Analyzer plug-in. The larger the node, the greater the degree value. There were 12 compounds with degrees ≥10. MOL008559 and MOL008569 were exceptions with no connected targets. The topological parameters of the major active compounds are shown in Table 2, suggesting that these 12 compounds may be the major active compounds of FT in treating HVR, including kaempferol, isorhamnetin, (2aR,2'S,4R,4'R,5'S,6aS,6bS,8aS,8bR,9S,11aR,12aR,12bR)-4,4'-dihydroxy-5',6a,8a,9-tetramethylicosahydro-1H-spiro [pentaleno[2,1-a]phenanthrene-10,2'-pyran]-8(2H)-one, (Z)-3-(4-hydroxy-3-methoxy-phenyl)-N- [2-(4-hydroxyphenyl)ethyl]acrylamide, terrestriamide, and tigogenin.

### 3.4. Construction and Analysis of the PPI Network

**Figure 3:**
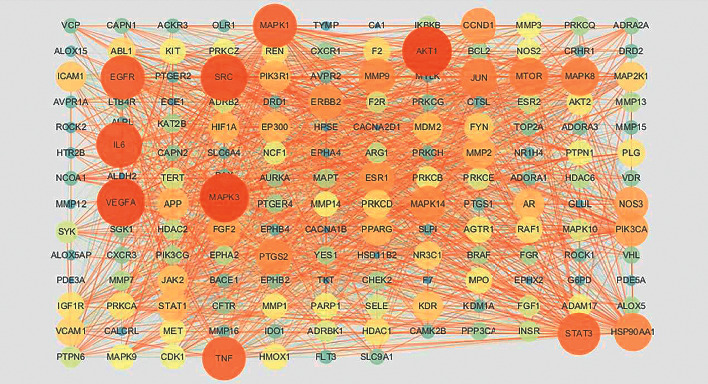
PPI network of FT in the treatment of HVR. The proteins with higher degrees are shown by larger nodes and brighter colors, and the edges with a greater combined score are shown by thicker and brighter lines.

The 164 common targets were uploaded to the STRING database to obtain the PPI network, and the results were saved as a TSV file and imported into Cytoscape software for processing to make the information more intuitive, as shown in Figure 3. The network diagram contained a total of 163 nodes and 2599 edges in which MERTK was not correlated with the other proteins and was, therefore, not included in the network. The proteins with greater degrees are shown as larger nodes and brighter color, and the edges with greater combined scores are shown by thicker and brighter lines. Targets whose betweenness centrality, closeness centrality, and degree all exceed the average values were selected as the major targets. The average degree in the network was 31.8896, the average closeness centrality was 0.5334, and the average betweenness centrality was 0.0056. Thirty-three main targets met the screening conditions. The specific information is shown in Table 3. Among them, AKT1 had the highest degree value, which can interact with 111 proteins, followed by MAPK3, VEGFA, SRC, and IL6, which can interact with 108, 106, 102, and 99 proteins, respectively.

**Figure 4:**
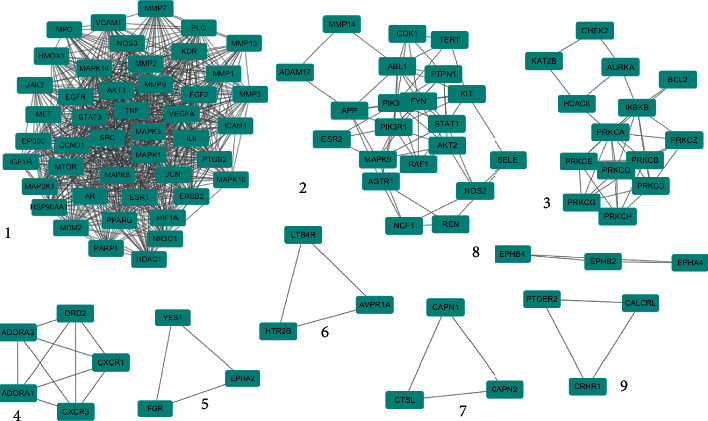
Clustering analysis results of the PPI network node. The blue nodes represent the targets, and the edges represent the interaction between them.

At the same time, the MCODE plug-in in Cytoscape software was used to analyze the PPI network. Nine significant clusters were obtained, as shown in Figure 4. Among them, the most significant, cluster 1, contained 45 major target nodes including the top targets in the PPI network, which further demonstrated the importance of AKT1, MAPK3, VEGFA, SRC, and IL6. The clustering details are shown in Table 4.

### 3.5. GO and KEGG Pathway Enrichment Analysis

**Figure 5:**
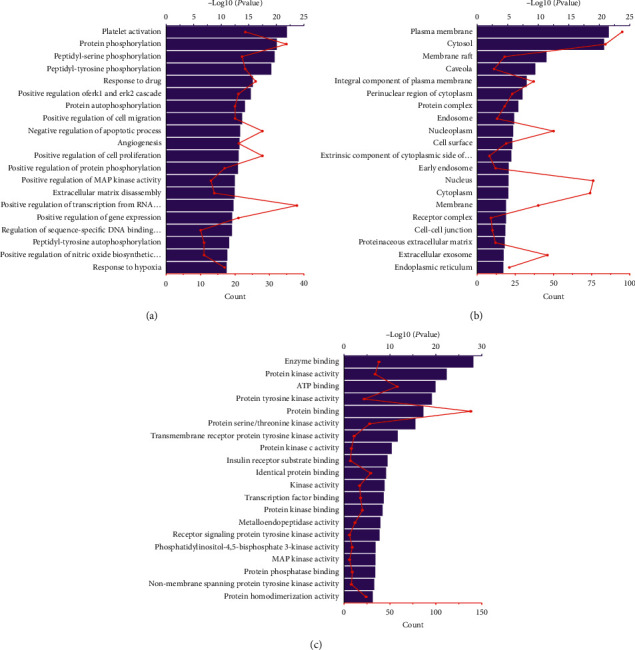
Results of GO enrichment analysis of the potential targets. (a) Biological processes. (b) Cellular components. (c) Molecular functions. The y-axis shows signiﬁcantly enriched categories of the targets and the x-axis shows the −log10 (*P* value) and enrichment counts of each term (*P* < 0.05).

The 164 common targets were inputted into the DAVID database for GO and KEGG analysis. Of them, 645 GO terms were ascertained, including 483 BP terms, 52 CC terms, and 110 MF terms. The top 20 enriched GO terms were partially displayed by a bar chart according to the −log10 (*P* value), and the count of each term is also displayed in Figure 5. The results showed that the BPs were correlated with platelet activation, protein phosphorylation, peptidyl-serine phosphorylation, etc. The major CCs included the plasma membrane, cytosol, membrane raft, etc. The targets of MF mainly involved enzyme-binding, protein kinase activity, and ATP binding.

**Figure 6:**
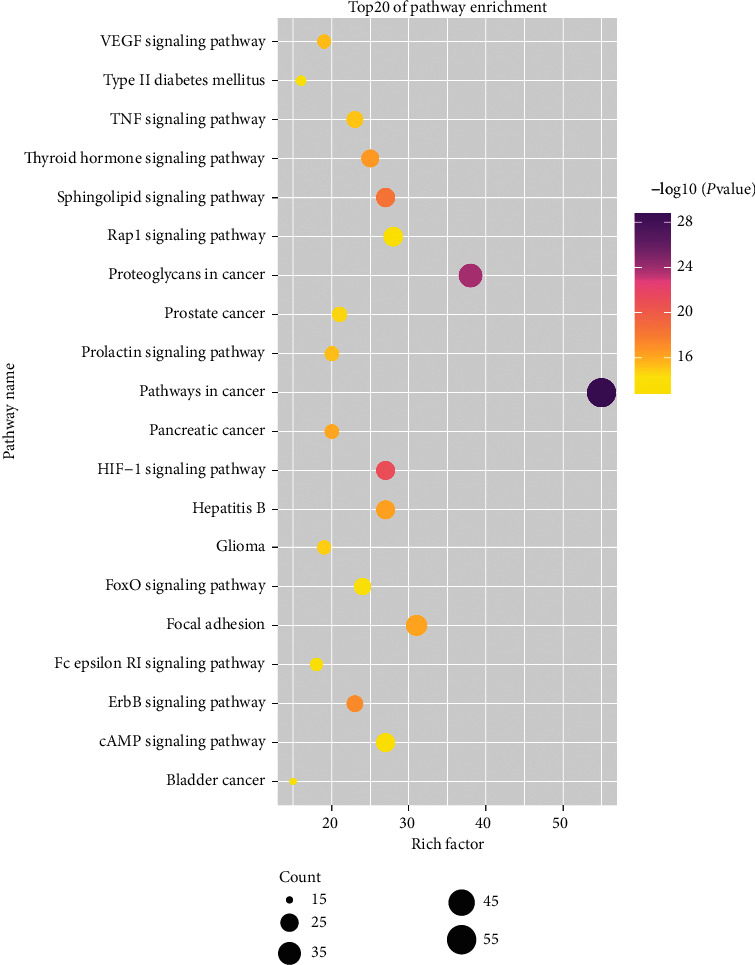
Results of KEGG enrichment analysis of the potential targets. The color of the nodes was determined by −log10 (*P* value) and the size of the nodes represents the number of counts (*P* value <0.05).

KEGG pathway annotation showed that the 164 common targets were involved in 122 pathways with *P* values of less than 0.05. The top 20 signaling pathways were listed in a bubble chart and are shown in Figure 6. The size of the dot reflects the number of targets in the analysis, and the different dot colors indicate the different *P* values. The enrichment pathways of FT in treating HVR were mainly concentrated in the HIF-1 signaling pathway, ErbB signaling pathway, VEGF signaling pathway, TNF signaling pathway, FoxO signaling pathway, Fc epsilon RI signaling pathway, Rap1 signaling pathway, and the cAMP signaling pathway.

### 3.6. Compound-Target-Pathway Network Analysis

**Figure 7:**
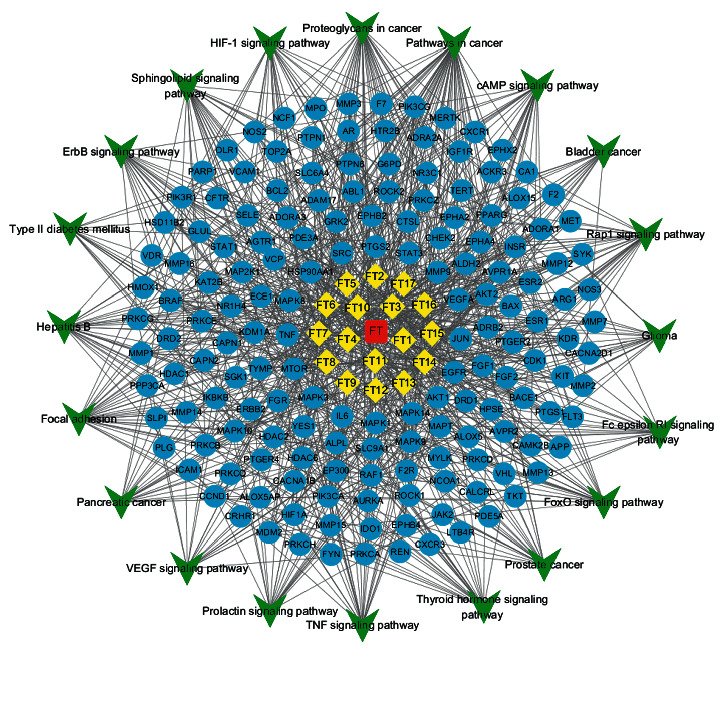
Compound-target-pathway network. The red nodes represent the drug. The yellow nodes represent the compounds. The blue nodes represent the targets. The green nodes represent the pathway of FT in the treatment of HVR. The edges represent the interaction between them.

The active compounds, potential targets, and signal pathways were imported into Cytoscape software for visual processing to construct a compound-target-pathway network.  Figure 7 shows that one active compound could correspond to multiple targets, and one target could correspond to multiple active compounds and multiple pathways. The network diagram fully reflected the characteristics of the synergistic relationships between the multiple components, targets, and pathways of FT.

### 3.7. Major Active Compound-Main Target Molecular Docking

**Figure 8:**
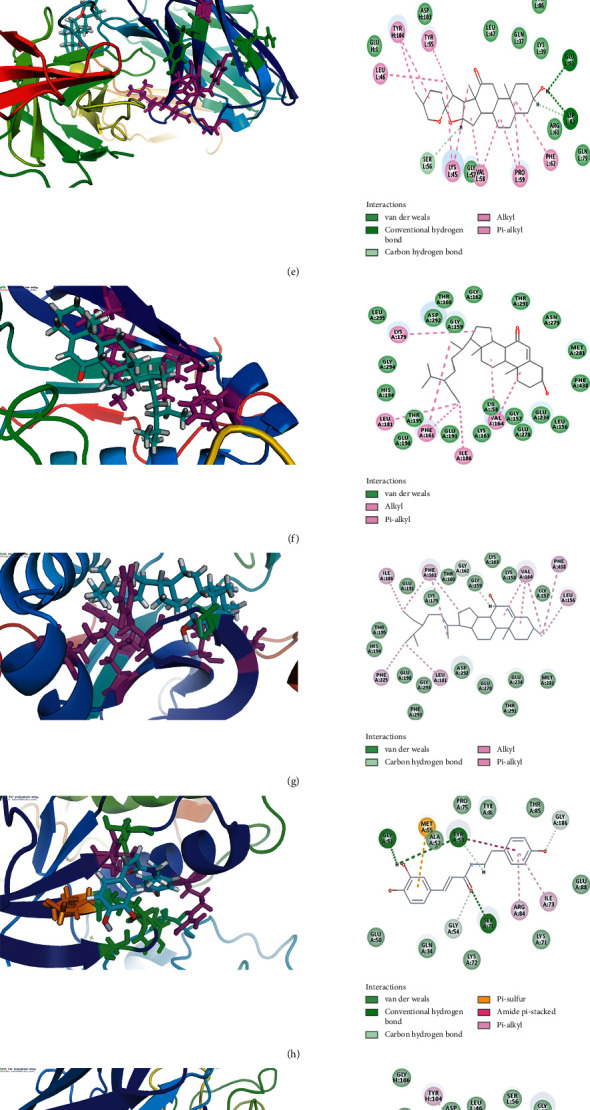
Molecular docking between the major active compounds and main targets. (a) FT3 docking with VEGFA. (b) FT1 docking with VEGFA. (c) FT11 docking with VEGFA. (d) FT4 docking with MAPK3. (e) FT10 docking with VEGFA. (f) FT14 docking with AKT1. (g) FT6 docking with AKT1. (h) FT8 docking with MAPK3. (i) FT13 docking with VEGFA. (j) FT7 docking with MAPK3. (k) FT2 docking with VEGFA. (l) FT15 docking with SRC.

To further analyze and verify the target-compound interactions, the top five main targets of AKT1, MAPK3, VEGFA, SRC, and IL6, which had higher degrees, were selected for molecular docking with the 12 major active compounds of FT. The LibDock score of the 12 small molecule compounds was obtained, as shown in Table 5. The component had a good affinity for the target when the LibDock score between the compound and protein was higher than the score between the ligand and protein. This table indicated that each of the 12 active compounds had one or more docking proteins with a high score, and VEGFA could bind to most compounds. The details of the docking simulation of the compound targets with higher docking scores are shown in Figure 8. In the two-dimensional schematic, the dotted line represents the interaction between the compound and the amino acid residues of the protein. The compound and the protein could form different types of interactions such as van der Waals, carbon-hydrogen bonds, conventional hydrogen bonds, and alkyl bonds. For example, when kaempferol bound to VEGFA, kaempferol interacted with TYR104 and PRO59 via carbon-hydrogen bonds, and kaempferol interacted with LEU47 and LYS45 to form a pi-alkyl interaction. Based on these data, we can consider that the interactions are the basis of their biologic activities.

## 4. Discussion

Hypertension is one of the most common cardiovascular diseases, with an increasing prevalence rate worldwide. Vascular remodeling is a significant pathological feature of hypertension, in which the early stage is an adaptive process that eventually becomes maladaptive, damaging target organs such as the heart, brain, and kidneys, and causing the complications of hypertension. TCM has a long history of treating hypertension, with the characteristics of maintaining stable hypotension and protecting target organs with fewer adverse reactions. FT has been used as a TCM for thousands of years and has a certain effect on the treatment of HVR. Although some preliminary research has been conducted on FT, the molecular targets and mechanisms of FT in treating HVR have not been fully explored. Therefore, elucidating the potential mechanisms is of great significance.

Network pharmacology is a new strategy and method, which helps to investigate the interaction between drugs and diseases and explores the underlying mechanism of a particular therapeutic efficacy. In this study, we constructed a compound-disease target interaction network and found 12 major active compounds. Meanwhile, three topological parameters, degree, betweenness centrality, and closeness certainty were calculated to identify the main targets in the PPI network. Then, GO and KEGG pathway enrichment analyses were carried out to further explore the potential mechanisms of FT in the treatment of HVR. At last, we used molecular docking methods to evaluate the binding activity between the targets and active compounds of FT.

Through the PPI network, we found 33 main target proteins of FT in treating HVR. Among them, the regulation of the MAPK target was of great significance in the treatment of HVR. MAPK mediates a large variety of biological functions including gene expression, cell mitosis, metabolism, motility, survival, apoptosis, and differentiation [35]. One study demonstrated that luteolin could exert effects on HVR by inhibiting angiotensin II-induced proliferation and the migration of vascular smooth muscle cells by regulating the MAPK pathway [36]. VEGFA is a proangiogenic factor [37] active in angiogenesis, vasculogenesis, and endothelial cell growth. It was the first member of the VEGF family to be discovered and is the most studied member. Related studies found that the downregulation of VEGFA protein levels could inhibit cell migration and the tube formation of human umbilical vein endothelial cells induced by conditioned medium derived from U251 cell culture [38]. Simultaneously, the VEGF/Flt-1 pathway could enhance the production of inflammatory molecules, chemotactic mediators, and adhesion molecules and induce vascular remodeling in an autocrine or paracrine manner. A previous study demonstrated that reduced VEGF protein expression and its receptor Flt-1 could decrease neovascularization and improve vascular remodeling [39–41]. IL6 is a pleiotropic cytokine and studies have demonstrated that IL-6 plays a crucial role in the pathophysiology of Takayasu arteritis and giant cell arteritis [42]. Takahiro et al. found that IL-6 blockade by a monoclonal anti-IL-6 receptor antibody could ameliorate hypoxia-induced pulmonary hypertension [43]. According to the literature [44], in supracoronary aortic banding plus metabolic syndrome animals, reducing IL-6, either by anti-IL-6 antibody or metformin treatment, could reverse pulmonary vascular remodeling.

The KEGG analysis provided a further understanding of the mechanism of HVR. HIF-1 is a transcription factor that regulates the homeostasis of oxygen concentration and is activated under hypoxic conditions, resulting in the upregulation of its target genes, which include VEGF. Many studies have confirmed that the HIF-1 signaling pathway is related to cardiovascular diseases. Mo et al. found that the onset of capillary leakage was associated with an upregulated HIF-1a/VEGFA signaling pathway, causing dysregulation in the immune response and affecting vascular permeability [45]. One cell culture study pointed to endothelial cell HIF signaling as important for connective tissue growth factor expression, vascular permeability, and endothelium-smooth muscle cell interactions that could promote vascular remodeling. In addition, the results demonstrated that endothelial HIF signaling could regulate pulmonary fibrosis-associated pulmonary hypertension [46]. The ErbB signaling pathway was another pathway enriched in our study. The NRG/ErbB system plays a crucial role in the development, maintenance of function, and repair responses to injury in the cardiovascular system. Hedhli et al. summarized that the ErbB signaling pathway is an important mediator of vascular preservation and angiogenic responses in the endothelium [47]. The VEGF signaling pathway fulfills a cardinal role in endothelial cells and its inhibition has profound cardiovascular impacts. TNF is a proinflammatory factor mainly secreted by macrophages. The TNF signaling pathway can participate in the systemic inflammatory response and interact with the NF-*κ*B and MAPK pathways, leading to the gradual progression of the disease. The FoxO signaling pathway has important roles in cell fate decisions including apoptosis, cell-cycle control, glucose metabolism, oxidative stress resistance, and longevity [48]. These results suggest that targets were involved in many related pathways, demonstrating the multiple compounds, targets, and pathway characteristics of FT in the treatment of HVR and providing a basis for understanding the molecular mechanisms involved.

## 5. Conclusion

Although the efficacy of FT in treating HVR has been confirmed by many studies, the molecular mechanisms of FT remain unclear. In the present study, we screened 12 major active compounds and 33 major targets. Functional enrichment analyses including GO and KEGG pathway analyses were performed, and 645 GO terms and 122 pathways were ascertained. Last, we used molecular docking methods to evaluate the binding activity between the targets and active compounds of FT. This study clarified the multiple compounds, targets, and pathway characteristics of FT in the treatment of HVR and provided a systematic view of the potential mechanisms from the combination of network pharmacology and a literature search.

## Figures and Tables

**Table 1:** Detailed information on 17 active compounds from FT.

Number	Molecule ID	Molecule name	Molecule weight	OB (%)	DL
FT1	MOL000354	Isorhamnetin	316.28	49.60	0.31
FT2	MOL000359	Sitosterol	414.79	36.91	0.75
FT3	MOL000422	Kaempferol	286.25	41.88	0.24
FT4	MOL000483	(Z)-3-(4-hydroxy-3-methoxy-phenyl)-N-[2-(4-hydroxyphenyl)ethyl]acrylamide	313.38	118.35	0.26
FT5	MOL008559	(2aR,2'R,4R,6aR,6bS,8aS,8bR,11aS,12aR,12bR)-4-((S)-2-(2,6-dimethylphenyl)propoxy)-5',5',6a,8a-tetramethyl-8-methylenedocosahydro-1H-spiro[pentaleno[2,1-a]phenanthrene-10,2'-pyran]	573.00	59.49	0.28
FT6	MOL008563	(3R,8S,9S,10R,13R,14R,17S)-17-((2S,5R)-5-ethyl-6-methylheptan-2-yl)-3-hydroxy-10,13-dimethyl-3,4,8,9,10,11,12,13,14,15,16,17-dodecahydro-1H-cyclopenta[a]phenanthren-7(2H)-one	428.77	40.93	0.79
FT7	MOL008567	(3R,7R,8S,9S,10S,13R,14S,17R)-17-((2R,5S)-5-ethyl-6-methylheptan-2-yl)-3,10-dimethyl-2,3,4,7,8,9,10,11,12,13,14,15,16,17-tetradecahydro-1H-cyclopenta[a]phenanthren-7-ol	414.79	34.21	0.76
FT8	MOL008568	(Z)-3-(3,4-dihydroxyphenyl)-N-[2-(4-hydroxyphenyl)ethyl]acrylamide	299.35	113.25	0.24
FT9	MOL008569	*β*-sitosterol-*β*-D-glucopyranoside	398.79	32.41	0.71
FT10	MOL008588	Terrestriamide	327.36	114.09	0.29
FT11	MOL008590	(2aR,2'S,4R,4'R,5'S,6aS,6bS,8aS,8bR,9S,11aR,12aR,12bR)-4,4'-dihydroxy-5',6a,8a,9-tetramethylicosahydro-1H-spiro[pentaleno[2,1-a]phenanthrene-10,2'-pyran]-8(2H)-one	444.72	58.74	0.76
FT12	MOL008593	(2aR,5S,6aS,6bS,8aS,8bS,11aS,12aR,12bR)-10-isopentyl-6a,8a,9-trimethyl-2,2a,3,4,5,6,6a,6b,7,8,8a,8b,11a,12,12a,12b-hexadecahydro-1H-naphtho[2',1':4,5]indeno[2,1-b]furan-5-ol	400.71	39.21	0.84
FT13	MOL007291	Hecogenin	430.69	15.86	0.79
FT14	MOL008581	Tigogenin	416.71	13.83	0.81
FT15	78177919	Terrestrosin D	1049.16	—	—
FT16	122169314	Terrestrosin K	1079.18	—	—
FT17	—	Terrestroside B	1312.63	—	—

**Table 2:** Topological parameters of the major active compounds in the compound-target network.

Number	Molecule ID	Betweenness centrality	Closeness centrality	Degree
FT3	MOL000422	0.23	0.43	57
FT1	MOL000354	0.17	0.42	50
FT11	MOL008590	0.19	0.42	47
FT4	MOL000483	0.14	0.41	44
FT10	MOL008588	0.14	0.40	41
FT14	MOL008581	0.14	0.40	41
FT6	MOL008563	0.14	0.40	37
FT8	MOL008568	0.12	0.39	33
FT13	MOL007291	0.07	0.38	30
FT7	MOL008567	0.03	0.35	14
FT2	MOL000359	0.02	0.36	13
FT15	78177919	0.09	0.35	13

**Table 3:** Topological parameters of the main targets of FT in the treatment of HVR.

Gene names	Protein names	Betweenness centrality	Closeness centrality	Degree
AKT1	RAC-alpha serine/threonine-protein kinase	0.07	0.75	111
MAPK3	Mitogen-activated protein kinase 3	0.06	0.75	108
VEGFA	Vascular endothelial growth factor A	0.06	0.73	106
SRC	Proto-oncogene tyrosine-protein kinase Src	0.04	0.73	102
IL6	Interleukin-6	0.05	0.71	99
EGFR	Epidermal growth factor receptor	0.04	0.70	94
MAPK1	Mitogen-activated protein kinase 1	0.03	0.70	93
TNF	Tumor necrosis factor	0.03	0.68	92
STAT3	Signal transducer and activator of transcription 3	0.02	0.68	88
MAPK8	Mitogen-activated protein kinase 8	0.01	0.64	75
MTOR	Serine/threonine-protein kinase mTOR	0.02	0.64	75
JUN	Transcription factor AP-1	0.01	0.64	74
HSP90AA1	Heat shock protein HSP 90-alpha	0.02	0.64	74
MMP9	Matrix metalloproteinase-9	0.01	0.62	69
MAPK14	Mitogen-activated protein kinase 14	0.01	0.62	68
PTGS2	Prostaglandin G/H synthase 2	0.01	0.62	68
CCND1	G1/S-specific cyclin-D1	0.01	0.62	67
ESR1	Estrogen receptor	0.01	0.61	64
ERBB2	Receptor tyrosine-protein kinase erbB-2	0.01	0.61	64
PIK3CA	Phosphatidylinositol 4,5-bisphosphate 3-kinase catalytic subunit alpha isoform	0.01	0.61	62
NOS3	Nitric oxide synthase, endothelial	0.02	0.62	62
FGF2	Fibroblast growth factor 2	0.01	0.60	59
PIK3R1	Phosphatidylinositol 3-kinase regulatory subunit alpha	0.01	0.59	55
PPARG	Peroxisome proliferator-activated receptor gamma	0.01	0.59	54
EP300	Histone acetyltransferase p300	0.01	0.59	54
STAT1	Signal transducer and activator of transcription 1-alpha/beta	0.01	0.59	53
APP	Amyloid-beta precursor protein	0.02	0.59	53
PRKCD	Protein kinase C delta type	0.01	0.56	44
NR3C1	Glucocorticoid receptor	0.02	0.57	43
F2	Prothrombin	0.01	0.56	41
PRKCA	Protein kinase C alpha type	0.01	0.56	41
HDAC1	Histone deacetylase 1	0.01	0.56	40
REN	Renin	0.01	0.56	38

**Table 4:** Clustering analysis results of the PPI network node.

Cluster	Genes	Score	Nodes	Edges
1	MDM2, EGFR, EP300, HDAC1, MPO, MAPK10, PLG, MMP9, SRC, MMP2, ERBB2, PPARG, MMP3, STAT3, CCND1, JAK2, PARP1, KDR, PTGS2, VEGFA, TNF, VCAM1, MTOR, MAP2K1, MMP1, NOS3, AKT1, AR, ICAM1, MAPK3, MAPK14, MMP7, FGF2, NR3C1, IGF1R, MAPK1, MAPK8, HMOX1, ESR1, HIF1A, JUN, IL6, MMP13, MET, HSP90AA1	34.182	45	752
2	NCF1, REN, NOS2, FYN, ADAM17, STAT1, PIK3CA, PIK3R1, KIT, CDK1, PTPN1, MMP14, SELE, TERT, RAF1, MAPK9, ABL1, AKT2, AGTR1, APP, ESR2	6.500	21	65
3	PRKCE, PRKCB, PRKCD, PRKCA, BCL2, PRKCQ, AURKA, PRKCG, CHEK2, HDAC6, KAT2B, PRKCH, PRKCZ, IKBKB	6.000	14	39
4	CXCR1, CXCR3, ADORA3, ADORA1, DRD2	5.000	5	10
5	CALCRL, PTGER2, CRHR1	3.000	3	3
6	LTB4R, AVPR1A, HTR2B	3.000	3	3
7	CTSL, CAPN2, CAPN1	3.000	3	3
8	EPHB4, EPHB2, EPHA4	3.000	3	3
9	CALCRL, PTGER2, CRHR1	3.000	3	3

**Table 5:** LibDock scores of major active compound-main target molecular docking.

Number	Compounds	LibDock Score				
		AKT1	MAPK3	VEGFA	SRC	IL6
FT3	Kaempferol	60.87	68.44	86.37	70.98	0.00
FT1	Isorhamnetin	60.50	112.15	123.48	92.79	67.24
FT11	(2aR,2'S,4R,4'R,5'S,6aS,6bS,8aS,8bR,9S,11aR,12aR,12bR)-4,4'-dihydroxy-5',6a,8a,9-tetramethylicosahydro-1H-spiro[pentaleno[2,1-a]phenanthrene-10,2'-pyran]-8(2H)-one	0.00	0.00	81.20	0.00	72.51
FT4	(Z)-3-(4-hydroxy-3-methoxy-phenyl)-N-[2-(4-hydroxyphenyl)ethyl]acrylamide	94.50	110.96	94.66	99.70	85.32
FT10	Terrestriamide	0.00	0.00	96.83	96.77	88.63
FT14	Tigogenin	123.52	104.95	118.77	81.80	79.52
FT6	(3R,8S,9S,10R,13R,14R,17S)-17-((2S,5R)-5-ethyl-6-methylheptan-2-yl)-3-hydroxy-10,13-dimethyl-3,4,8,9,10,11,12,13,14,15,16,17-dodecahydro-1H-cyclopenta[a]phenanthren-7(2H)-one	127.07	62.24	116.71	110.59	83.07
FT8	(Z)-3-(3,4-dihydroxyphenyl)-N-[2-(4-hydroxyphenyl)ethyl]acrylamide	90.15	107.96	93.94	89.87	88.15
FT13	Hecogenin	0.00	0.00	76.87	0.00	0.00
FT7	(3R,7R,8S,9S,10S,13R,14S,17R)-17-((2R,5S)-5-ethyl-6-methylheptan-2-yl)-3,10-dimethyl-2,3,4,7,8,9,10,11,12,13,14,15,16,17-tetradecahydro-1H-cyclopenta[a]phenanthren-7-ol	95.34	113.95	94.71	87.17	92.55
FT2	Sitosterol	0.00	0.00	108.59	55.55	61.00
FT15	Terrestrosin D	0.00	0.00	151.75	167.11	0.00
—	Ligand	86.31	88.48	47.65	133.52	72.78

## Data Availability

All data generated or analyzed during this study are included within the paper.

## Abbreviations

ADME: Absorption, distribution, metabolism, and excretion
BP: Biological processes
CC: Cellular components
DL: Drug-likeness
FT: Fructus Tribuli
GO: Gene ontology
HVR: Hypertensive vascular remodeling
KEGG: Kyoto encyclopedia of genes and genomes
MCODE: Molecular complex detection
MF: Molecular functions
OB: Oral bioavailability
OMIM: Online Mendelian Inheritance in Man
PPI: Protein-protein interaction network
SHRs: Spontaneously hypertensive rats
TCM: Traditional Chinese medicine
TCMSP: Traditional Chinese Medicine Systems Pharmacology Database and Analysis Platform
TSV: Tab-separated value.
